# Mitochondria-associated endoplasmic reticulum membranes in canine and feline diabetic kidney disease: mechanistic links and nutritional implications

**DOI:** 10.3389/fvets.2026.1848289

**Published:** 2026-09-02

**Authors:** Qingwen Yang, Yu Sun, Li Zuo, Maosen Xu, Suwen Tan, Zhihao Wang, Mingzheng Han

**Affiliations:** 1 Laboratory of Veterinary Pharmacology, Department of Animal Science and Technology, Chongqing Three Gorges Vocational College, Chongqing, China; 2 College of Veterinary Medicine, South China Agricultural University, Guangzhou, China

**Keywords:** diabetes mellitus, food development, functional therapeutic diets, kidney disease, mitochondria-associated endoplasmic reticulum membrane

## Abstract

Diabetic kidney disease in dogs and cats is clinically relevant but mechanistically underdefined, and the dietary management of diabetic pets is still dominated by empirical control of glycemia, body weight, and renal nutrient load. Mitochondria-associated endoplasmic reticulum membranes (MAMs) are dynamic contact domains that coordinate calcium transfer, lipid exchange, ER stress, mitochondrial quality control, inflammatory signaling, and cell death. This review first summarizes the veterinary clinical context of canine and feline diabetic kidney disease, including proteinuria, microalbuminuria, hypertension, chronic kidney disease, and species-specific differences in diabetes pathophysiology. It then integrates MAM biology into core renal injury modules involving structural tethering proteins, the IP3R-GRP75-VDAC-MCU/MICU1 calcium axis, lipid metabolic enzymes, PERK/GRP78-mediated ER stress, mPTP opening, mitophagy, and inflammatory amplification. Because direct MAM evidence in diabetic dogs and cats remains limited, the MAM-related pathways discussed here are presented as hypotheses inferred from rodent DKD models, human cell lines, and broader metabolic-disease studies rather than as confirmed mechanisms in companion animals. Finally, we discuss how MAM-centered mechanisms may guide the development of functional prescription diets incorporating EPA/DHA, medium-chain triglycerides, L-carnitine, taurine, magnesium, N-acetylcysteine, antioxidants, resveratrol, and selected plant extracts, provided that efficacy and safety are validated using species-specific renal endpoints. This perspective may support a transition from empirical formulation to mechanism-informed precision nutrition for diabetic dogs and cats.

## 1 Introduction

Diabetes mellitus is a clinically important endocrinopathy of dogs and cats, but the disease phenotype, therapeutic priorities, and complication profile differ substantially between the two species. Dogs most often present with insulin-dependent diabetes, whereas feline diabetes more frequently develops on a background of obesity-associated insulin resistance, beta-cell dysfunction, and variable potential for remission. Contemporary clinical guidelines therefore recommend species-specific diagnostic and therapeutic reasoning rather than treating canine and feline diabetes as a single entity (1, 2).

Kidney involvement is increasingly recognized as a relevant but incompletely defined complication in companion-animal diabetes. In dogs with spontaneous diabetes, longitudinal clinical studies have reported proteinuria, hypertension, and retinopathy as important comorbid features requiring monitoring (3). Canine urine proteomic analyses further suggest that renal molecular alterations may be detectable before or around the onset of microalbuminuria (4). In cats, population-level data support an association between diabetes mellitus and chronic kidney disease (CKD), while histopathological and ultrastructural studies have demonstrated glomerular changes compatible with early diabetic nephropathy (DN) in subsets of diabetic cats (5–7).

Despite these observations, the mechanistic framework linking metabolic disease to renal injury in dogs and cats remains less mature than in human and rodent diabetic kidney disease (DKD). This gap is particularly relevant for the Animal Nutrition and Metabolism section because prescription diets for diabetic pets are frequently designed around glycemic control, body weight, and renal-protective macronutrient constraints, whereas organelle-level mechanisms are rarely integrated into formulation logic. Mitochondria-associated endoplasmic reticulum membranes (MAMs) offer a biologically coherent bridge between diabetic metabolic stress, renal cellular injury, and functional ingredient discovery.

MAMs are specialized membrane contact domains through which the endoplasmic reticulum (ER) and mitochondria exchange calcium, lipids, metabolites, and stress signals. Their resident or regulatory proteins include structural tethers such as MFN2 and PACS-2, calcium-transfer components such as IP3R, GRP75, VDAC, MCU, and MICU1, lipid metabolic enzymes such as ACSL4, ACAT, DGAT2, and PISD, and stress-response or quality-control proteins such as PERK, GRP78, PINK1, Parkin, and mTOR-related signaling nodes (8–11). This review is structured to move from veterinary clinical context to MAM biology, core renal pathways, and cautious companion-animal diet development.

## 2 Veterinary clinical context: canine and feline diabetic kidney disease

### 2.1 Diabetes phenotypes differ between dogs and cats

The first translational constraint is that canine and feline diabetes are not equivalent biological syndromes. Canine diabetes is commonly characterized by persistent insulin requirement and is influenced by breed, sex, pancreatitis, diestrus, immune-mediated beta-cell loss, and other endocrine disorders (12, 13). Feline diabetes more often resembles type 2 diabetes in humans, with obesity, reduced insulin sensitivity, islet amyloid deposition, glucotoxicity, and the possibility of diabetic remission after effective management (2, 13, 14). In this review, DN is used to denote the classical glomerular pathological changes (e.g., glomerular basement membrane thickening, mesangial expansion, and nodular sclerosis), whereas DKD is adopted as a broader term that encompasses injury to all renal compartments, including tubular, interstitial, and vascular lesions, consistent with current clinical and experimental nomenclature.

These differences affect how renal mechanisms should be interpreted. In dogs, chronic insulin deficiency and glycemic volatility may dominate the metabolic pressure placed on renal cells. In cats, obesity, insulin resistance, dyslipidemia, and concurrent CKD may be more prominent drivers. Therefore, MAM-related claims in this manuscript are framed as candidate mechanistic pathways for validation rather than as established species-level conclusions unless direct canine or feline data are available.

### 2.2 Renal phenotypes relevant to diabetic dogs and cats

In companion-animal clinical practice, diabetic kidney involvement is usually recognized through combinations of proteinuria or microalbuminuria, altered urine specific gravity, systemic hypertension, changes in creatinine or symmetric dimethylarginine (SDMA), reduced glomerular filtration, and evidence of CKD. These endpoints do not map perfectly onto the classic human DKD trajectory, and histological confirmation is uncommon in routine veterinary practice. Nevertheless, the available evidence justifies closer attention to renal monitoring in diabetic pets.

In dogs, a longitudinal study of spontaneous diabetes documented the development of hypertension, proteinuria, and retinopathy over time, indicating that diabetic dogs may develop clinically relevant microvascular complications (3). Urinary proteomic studies have identified differences among healthy dogs, diabetic dogs without microalbuminuria, and diabetic dogs with microalbuminuria, supporting the possibility of early renal molecular remodeling before overt renal dysfunction (4). In cats, diabetes has been associated with CKD after adjustment for age, and renal morphology studies have described glomerular basement membrane thickening and other glomerular or vascular alterations in diabetic cats (5–7).

### 2.3 Clinical phenotypic combinations that should guide mechanistic interpretation

For dogs and cats with diabetes, renal injury rarely occurs in isolation. Relevant clinical combinations include diabetic ketoacidosis or ketosis, recurrent urinary tract infection, pancreatitis, obesity or sarcopenia, hypertension, retinopathy, hyperlipidemia, CKD, and dietary constraints arising from concurrent renal disease. These combinations are important because MAM biology is sensitive to nutrient availability, oxidative stress, ER stress, lipids, and inflammatory tone (15, 16). A formulation that is mechanistically attractive in an obese insulin-resistant cat may be inappropriate for a dog with advanced CKD, poor body condition, and phosphorus restriction. This review therefore treats MAM-directed nutrition as an adjunctive and individualized strategy rather than as a single universal dietary formula.

## 3 MAM architecture and renal-cell functions relevant to diabetes

### 3.1 Structural organization and basis for inference

MAMs are not fixed organelles but dynamic contact domains formed where ER and mitochondrial membranes are closely apposed. Electron microscopy and functional calcium-imaging studies have demonstrated that the distance and stability of ER-mitochondrial contacts are critical for localized calcium transfer, lipid exchange, mitochondrial metabolism, and stress signaling (8, 17, 18). Excessive separation may weaken metabolic coupling, whereas excessive or maladaptive tightening may promote calcium overload and mitochondrial injury. Thus, the biological objective is not simply to increase or decrease MAM formation, but to restore appropriate MAM dynamics in a specific cell type and disease context.

Direct evidence on MAMs in canine and feline DN is still scarce. Therefore, this review discusses MAM-related mechanisms in companion animals mainly as hypotheses inferred from rodent DKD models, human cell lines, and broader metabolic-disease studies, while using dog and cat studies to define the clinical and nutritional context. These mechanisms should be tested in canine and feline renal models before they are used to support specific dietary recommendations.

### 3.2 Structural tethering proteins: MFN2, PACS-2, and related contact regulators

MFN2 is a central regulator of mitochondrial fusion and ER-mitochondrial apposition. In metabolic disease models, MFN2 links mitochondrial and ER function to insulin signaling and cellular energy homeostasis (19–21). In high-glucose podocyte models, MFN2-dependent MAM disruption has been associated with activation of the PERK pathway and apoptosis, suggesting a plausible link between MAM destabilization and glomerular cell injury (22). PACS-2 is another regulator of ER-mitochondrial coupling. In diabetic mouse kidneys and high-glucose tubular epithelial cells, PACS-2 deficiency aggravated proteinuria, renal dysfunction, ER stress, mitochondrial injury, apoptosis, and fibrosis, whereas PACS-2 restoration helped stabilize MAMs and attenuate tubular injury (23). A recent DKD study further showed that C5aR2 activation promoted phosphatidylserine synthase-MFN2 interaction and MAM formation, thereby improving phosphatidylserine metabolic homeostasis and protecting against diabetic kidney injury in experimental models (24).

For veterinary translation, these findings suggest that MFN2/PACS-2-centered MAM integrity could be examined in canine and feline renal tubular cells, podocytes, and diabetic kidney tissue. However, no direct evidence currently establishes that dietary ingredients restore MFN2 or PACS-2 in diabetic dogs or cats. Nutritional discussion is therefore restricted to the hypothesis that ingredients supporting mitochondrial quality control or reducing ER stress may indirectly preserve MAM function.

### 3.3 Calcium-transfer machinery: IP3R-GRP75-VDAC-MCU/MICU1 axis

A key function of MAMs is the transfer of calcium from ER stores to mitochondria. The canonical pathway involves IP3R on the ER membrane, GRP75 as a physical linker, VDAC on the outer mitochondrial membrane, and MCU/MICU1-dependent entry into the mitochondrial matrix (25–27). Physiological calcium transfer supports mitochondrial dehydrogenase activity and ATP production, but sustained or excessive calcium entry can trigger mitochondrial permeability transition pore opening, ROS production, bioenergetic collapse, and cell death (28, 29).

In diabetic renal cells, calcium dyshomeostasis is a plausible convergence point between glucotoxicity, ER stress, mitochondrial dysfunction, and podocyte or tubular injury. In dogs and cats, the direct clinical readouts are not cellular calcium fluxes but renal endpoints such as albuminuria, proteinuria, blood pressure, creatinine, SDMA, urine specific gravity, and progression of CKD. Future companion-animal studies should link these clinical endpoints with renal-cell readouts of MAM calcium machinery.

### 3.4 Lipid exchange and metabolic signaling at MAMs

MAMs also coordinate lipid synthesis, remodeling, and transfer. Enzymes such as ACSL4/FACL4, ACAT, DGAT2, and PISD support fatty-acid activation, cholesterol esterification, triacylglycerol synthesis, and phospholipid remodeling (15, 30, 31). In diabetes, dyslipidemia and ectopic lipid deposition can alter membrane composition, induce ER stress, disturb insulin signaling, and amplify mitochondrial ROS production. These mechanisms are particularly relevant to obese insulin-resistant cats and overweight diabetic dogs, but the exact lipid species driving renal injury may differ by species, diet, and comorbidity.

### 3.5 ER stress, mitophagy, and inflammatory signaling

Mitochondria-associated endoplasmic reticulum membranes are positioned at the intersection of ER stress, mitochondrial quality control, and inflammatory signaling. During ER stress, GRP78 dissociation, PERK-eIF2α-ATF4/CHOP activation, and calcium release can propagate injury toward mitochondria (32, 33). In parallel, PINK1/Parkin-mediated mitophagy and mTOR-regulated autophagy influence whether damaged mitochondria are removed or allowed to fuel oxidative stress and inflammation (34–36). Proper mitochondrial function relies on continuous protein import and folding (37); disruption of this process amplifies oxidative stress and cell death. Mitochondrial oxidative protein folding, catalyzed by enzymes such as Mia40 (38), is particularly vulnerable to diabetic redox imbalance. MAM disruption may also amplify NF-κB- and inflammasome-related responses, creating a feed-forward loop between ER stress, mitochondrial damage, and renal inflammation (16, 39, 40).

## 4 Core MAM-centered pathways in diabetic kidney injury

### 4.1 Metabolic overload and maladaptive MAM remodeling

The diabetic milieu exposes renal cells to hyperglycemia, altered insulin signaling, dyslipidemia, and increased oxidative stress. These stimuli can alter MAM tethering, calcium exchange, and lipid transfer. To avoid treating every MAM protein as an isolated target, these mechanisms can be organized into functional modules. The first module is maladaptive remodeling of ER-mitochondrial contact itself, involving MFN2, PACS-2, and possibly VAPB-PTPIP51-like tethering networks. In kidney disease models, either loss of protective contact integrity or excessive stress-induced coupling may be harmful depending on the cell type and stimulus.

### 4.2 Calcium overload, mitochondrial dysfunction, and cell death

Under persistent diabetic stress, excessive IP3R-driven ER calcium release and VDAC/MCU-dependent mitochondrial uptake may cause calcium overload, mPTP opening, mitochondrial depolarization, ATP decline, and ROS production. These events can impair podocyte cytoskeletal integrity, promote tubular epithelial injury, and contribute to fibrosis. Mitochondria are central integrators of cell death signals, and their dysfunction determines the switch between survival and death (41). MICU1 is especially relevant because it gates the threshold for mitochondrial calcium uptake; reduced MICU1 function can lower the threshold for overload. This pathway offers a concrete mechanistic axis for future canine/feline validation: IP3R-GRP75-VDAC1 interactions, MCU/MICU1 expression, mitochondrial calcium load, and mPTP susceptibility should be examined in renal cells exposed to diabetic and lipotoxic conditions.

### 4.3 ER stress-PERK-CHOP signaling and renal fibrosis

Chronic glucotoxicity, oxidative stress, and lipid perturbation can activate the unfolded protein response. In renal cells, maladaptive PERK-eIF2α-ATF4/CHOP signaling is associated with apoptosis, inflammatory activation, and fibrotic remodeling. MFN2 and PACS-2 studies suggest that preserving MAM integrity may attenuate ER stress under high-glucose conditions (22, 23). For companion animals, this module is clinically relevant because tubular injury and interstitial fibrosis are major determinants of CKD progression, but direct dog/cat evidence is still limited.

### 4.4 Lipotoxicity and membrane stress

In insulin-resistant states, altered fatty acid flux and lipid species accumulation can modify ER membrane composition, perturb phospholipid remodeling, and increase mitochondrial substrate stress. MAM-localized lipid enzymes may therefore influence both renal energy handling and susceptibility to lipid peroxidation. This issue is particularly important for nutritional design because changing dietary fat type, chain length, and omega-3/omega-6 balance can alter the lipidome. However, extrapolation from general lipid metabolism to renal MAM protection requires direct testing in canine and feline models.

### 4.5 Mitophagy, inflammation, and maladaptive repair

Adequate mitophagy can remove damaged mitochondria and limit ROS-dependent inflammatory signaling, whereas insufficient or excessive autophagy may worsen energy deficiency or tissue remodeling. Mitochondrial function depends on coordinated biogenesis and assembly of hundreds of proteins (42), and MAMs provide a critical platform for regulating this process. PINK1/Parkin and mTOR-related pathways therefore connect MAM integrity with renal stress adaptation (35, 43). In diabetic kidneys, failure to maintain mitochondrial quality may reinforce inflammatory cytokine production, inflammasome activation, apoptosis, and fibrosis. Future veterinary studies should define whether diabetic dogs and cats show altered mitophagy markers in urine, blood, or renal tissue and whether these changes track with proteinuria, SDMA, or CKD progression.

## 5 Species-specific interpretation for dogs and cats

### 5.1 Basis for mechanistic interpretation

Because direct MAM studies in diabetic dogs and cats remain scarce, this review does not present MAM-directed nutrition as an established treatment for companion-animal DN. Instead, it uses findings from rodent DKD models, human cell systems, and broader metabolic research to outline MAM-related pathways that may be involved in canine and feline DN. Clinical statements are limited to available dog/cat studies, whereas MAM-centered mechanisms are described as hypotheses requiring validation in veterinary renal cells, tissues, and feeding studies.

### 5.2 Canine considerations

In dogs, dietary development must account for insulin dependence, breed and sex predispositions, frequent cataracts or retinopathy, possible pancreatitis, risk of urinary tract infection, body-condition heterogeneity, and renal-protective constraints when CKD or proteinuria is present. Because dogs may present with proteinuria and hypertension during spontaneous diabetes, renal endpoints should be integrated into diabetic diet trials rather than assessed only after overt CKD develops (3). A canine MAM-directed diet should therefore be tested alongside standard insulin therapy and should include glycemic, renal, blood-pressure, body-composition, and safety endpoints.

### 5.3 Feline considerations

In cats, obesity-associated insulin resistance, potential remission, hypersomatotropism, concurrent CKD, and obligate carnivore nutritional requirements complicate diet design. Taurine adequacy, protein quality, carbohydrate restriction, phosphorus control, and palatability are all important. The coexistence of diabetes and CKD can create tension between diets designed for glycemic control and diets designed for renal protection. In such cases, clinical priority should be individualized; renal protection may require careful phosphorus control and high-quality protein selection, whereas glycemic control may require carbohydrate moderation and weight management.

## 6 MAM-directed prescription diet development for diabetic dogs and cats

### 6.1 General principles

**Figure 1:**
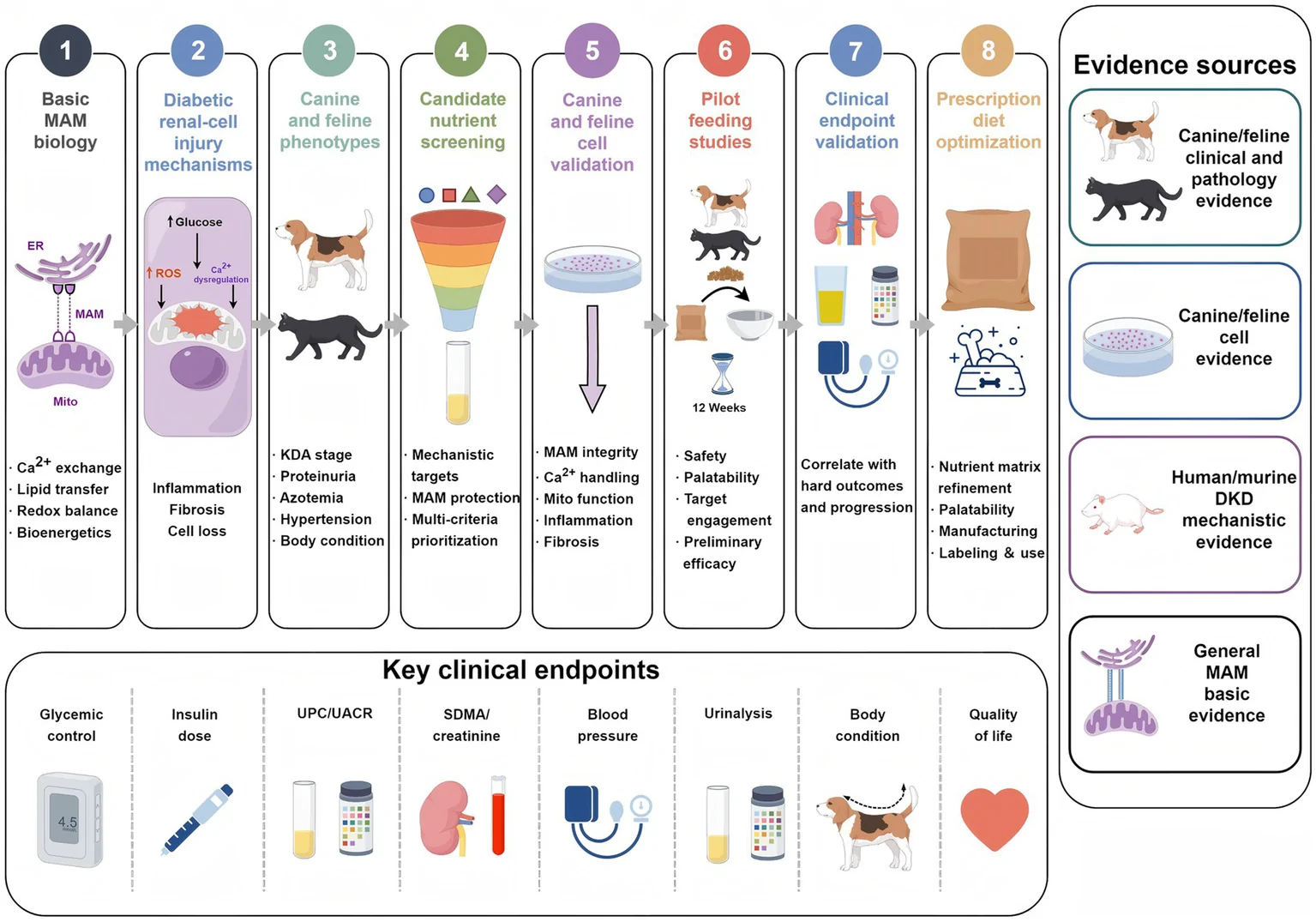
Proposed roadmap for MAM-directed nutrition in diabetic dogs and cats.

**Figure 2:**
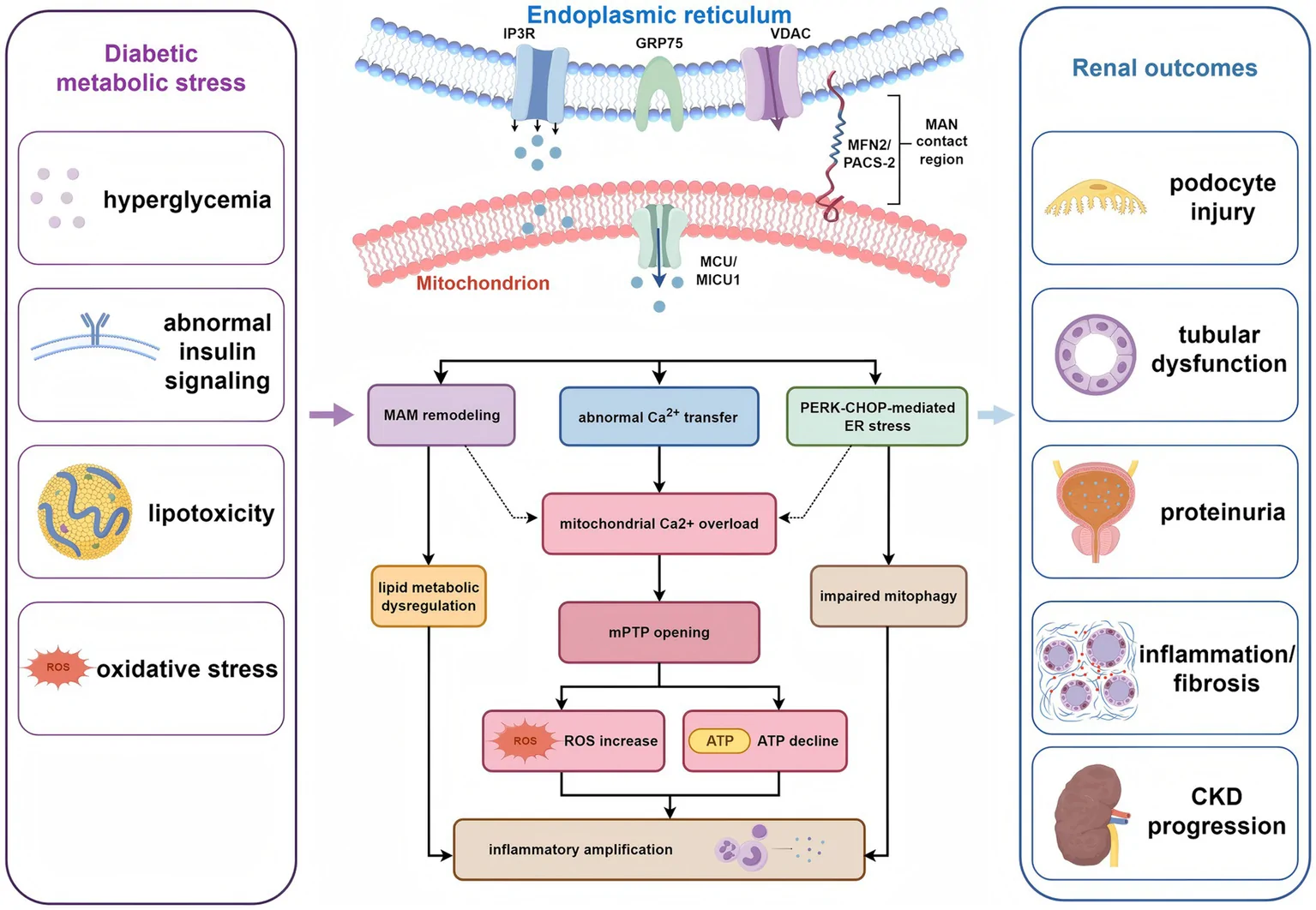
Proposed MAM-centered pathogenic modules in canine and feline diabetic kidney disease. The diagram depicts our proposed working model, in which diabetic metabolic stress may alter ER-mitochondrial contact zones, calcium flux, lipid metabolism, ER stress, mitochondrial dysfunction, mitophagy, and inflammation, leading to podocyte injury, tubular dysfunction, proteinuria, and CKD progression.

MAM-directed nutrition should be viewed as adjunctive to standard medical management, not as a substitute for insulin or other indicated therapy. Its goal is to support renal-cell bioenergetics, reduce ER-mitochondrial stress, improve lipid handling, and attenuate oxidative and inflammatory injury while maintaining species-appropriate nutrient balance. Figure 1 summarizes the evidence-to-application roadmap, and Figure 2 summarizes the proposed MAM-centered mechanisms that connect diabetic stress to renal injury.

A rational development program should include: (i) candidate ingredient selection based on MAM-relevant mechanisms and veterinary safety; (ii) validation in canine and feline renal cells under hyperglycemic, lipotoxic, and inflammatory conditions; (iii) pilot feeding studies assessing palatability, glycemic control, renal safety, lipidome changes, and oxidative-stress markers; and (iv) controlled clinical studies in diabetic dogs and cats with predefined renal endpoints.

### 6.2 Candidate functional components

Omega-3 long-chain polyunsaturated fatty acids. EPA and DHA may reduce inflammatory tone, modify membrane lipid composition, and support lipid mediator balance. In veterinary nutrition, omega-3 fatty acids have been studied in dogs and are widely considered relevant to inflammatory and renal conditions (44). However, omega-3 supplementation should consider dose, fat tolerance, oxidative stability, vitamin E balance, pancreatitis risk, and total dietary fat load. In a MAM framework, omega-3 fatty acids should be tested for their effects on ER calcium release, mitochondrial ROS, and inflammatory signaling in canine and feline renal models rather than assumed to directly regulate IP3R in pets.

Medium-chain triglycerides and L-carnitine. MCTs provide a more readily oxidized energy substrate than long-chain triglycerides and may influence circulating energetic lipids. In cats, DHA-enriched fish oil combined with MCTs increased ketone-body production and reduced N-acyl taurines and microbiome-derived putrefactive postbiotics (45). In dogs, fish oil and MCT feeding altered structural and energetic lipid profiles (46). These findings support further evaluation of MCTs and L-carnitine in diabetic dogs and cats, but renal MAM outcomes remain to be measured.

Taurine, magnesium, and ER/calcium homeostasis. Taurine is particularly important in cats and has broader roles in osmoregulation, antioxidant defense, and calcium handling (47, 48). Magnesium is relevant to cellular calcium antagonism and insulin signaling. These nutrients may be prioritized when the target mechanism is ER stress or calcium dyshomeostasis, but dosing should respect species-specific requirements and renal status.

Antioxidants, NAC, resveratrol, and polyphenols. Oxidative stress is a downstream consequence of mitochondrial dysfunction and a driver of renal inflammation. NAC, vitamin C, vitamin E, resveratrol, and selected plant polyphenols may protect redox balance or stress responses. A canine diabetic nephropathy study reported that NAC combined with insulin alleviated FUNDC1-mediated mitophagy dysregulation by regulating mitochondrial dynamics (49). *Plantago asiatica* and *Lonicera japonica* extracts have been investigated in canine and feline kidney cell models for anti-inflammatory effects (50). These data support cautious prioritization but do not yet prove clinical efficacy in diabetic pets with DN.

Autophagy-related nutrients. Spermidine and resveratrol have been discussed as autophagy or mitophagy modulators in metabolic and renal disease contexts (51, 52). In prescription diet development, these candidates should be evaluated for safety, palatability, interactions with medications, and the possibility that excessive autophagy could be harmful in animals with poor body condition or advanced CKD.

### 6.3 Proposed validation endpoints

Future studies should pair nutritional outcomes with mechanistic endpoints. Clinical endpoints should include insulin dose stability, continuous glucose monitoring or fructosamine, body condition, muscle condition, serum lipids, creatinine, SDMA, cystatin C when available, urine protein-to-creatinine ratio (UPC), urine albumin-to-creatinine ratio (UACR), blood pressure, urinalysis, and quality-of-life metrics. Mechanistic endpoints should include ER stress markers (GRP78, PERK, eIF2α, CHOP), mitochondrial function, ROS, mPTP sensitivity, mitophagy markers (PINK1, Parkin, LC3-II, p62), calcium-handling proteins (IP3R, VDAC1, MCU, MICU1), and MAM-contact readouts such as proximity ligation or electron microscopy in experimental models.

## 7 Outstanding questions and future directions

Several questions remain unresolved. First, it is unknown whether MAM structure is consistently reduced, increased, or dynamically remodeled in diabetic kidney tissue from dogs and cats. Second, podocytes, mesangial cells, endothelial cells, and tubular epithelial cells may respond differently to the same metabolic stimulus. Third, dietary ingredients that improve systemic metabolic status may not necessarily act directly on renal MAMs. Fourth, the timing of intervention may be critical; early microalbuminuria, overt proteinuria, and advanced CKD likely represent different therapeutic windows. Finally, future work should integrate metabolomics, lipidomics, urine proteomics, renal-cell biology, and controlled feeding studies to move from plausible mechanism to clinically useful diet design.

A practical research agenda would include: (i) defining MAM-contact markers in canine and feline renal cells; (ii) developing hyperglycemic and lipotoxic renal-cell models from both species; (iii) testing candidate nutrients individually and in combination; (iv) validating non-invasive biomarkers in diabetic pets; and (v) designing prospective feeding trials stratified by species, obesity status, glycemic control, proteinuria, and CKD stage.

## 8 Conclusion

MAMs provide a mechanistically coherent platform linking diabetic metabolic stress to renal mitochondrial dysfunction, ER stress, lipid dysregulation, inflammation, mitophagy disturbance, and cell death. Direct veterinary evidence remains limited, and human or rodent DKD findings should be translated cautiously. For canine and feline diabetes, MAM-directed nutrition is most appropriately viewed as a hypothesis-driven development strategy: candidate nutrients such as EPA/DHA, MCTs, L-carnitine, taurine, magnesium, NAC, antioxidants, resveratrol, and selected plant extracts should be tested through species-specific renal models and clinically relevant endpoints. This approach may support the evolution of diabetic pet prescription diets from empirical macronutrient adjustment toward mechanism-informed precision nutrition, while preserving the clinical priorities of glycemic control, renal protection, safety, and palatability.
